# Using machine learning to extract cognitive status from the sleep EEG in progressing stages of dementia: defining interpretable and age-related features

**DOI:** 10.1093/sleep/zsac324

**Published:** 2022-12-31

**Authors:** Korey Kam, Ankit Parekh, Sajila Wickramaratne, Andrew W Varga

**Affiliations:** Mount Sinai Integrative Sleep Center, Division of Pulmonary, Critical Care, and Sleep Medicine, and Friedman Brain Institute, Icahn School of Medicine at Mount Sinai, New York, NY, USA; Mount Sinai Integrative Sleep Center, Division of Pulmonary, Critical Care, and Sleep Medicine, and Friedman Brain Institute, Icahn School of Medicine at Mount Sinai, New York, NY, USA; Mount Sinai Integrative Sleep Center, Division of Pulmonary, Critical Care, and Sleep Medicine, and Friedman Brain Institute, Icahn School of Medicine at Mount Sinai, New York, NY, USA; Mount Sinai Integrative Sleep Center, Division of Pulmonary, Critical Care, and Sleep Medicine, and Friedman Brain Institute, Icahn School of Medicine at Mount Sinai, New York, NY, USA

Emerging evidence supports the bidirectional nature between sleep and Alzheimer’s disease (AD), such that sleep disturbances occurring in cognitively normal older individuals may accelerate the accumulation of AD pathology, and at the same time, AD pathology can change underlying features of sleep [1, 2]. Much of this work has taken the approach of distilling the polysomnogram (PSG) to a few variables in order to identify relationships between sleep macro- and microarchitecture features and clinical diagnoses and/or biomarkers of AD. An alternative approach is to mine the PSG data for a multitude of features and train machine learning algorithms to automatically classify disease states such as mild cognitive impairment (MCI) and dementia.

In this issue of SLEEP, Ye et al. [3] train and characterize algorithms that use EEG-derived sleep macro- and microarchitecture features from the PSG to predict clinical diagnoses of MCI and dementia. Among close to 11 000 clinical PSG’s to which the authors had access, approximately 1000 features were computed and used to train machine learning models such as random forest, support vector machine, and simple logistic regression on clinical diagnoses of dementia or MCI as subjectively defined from the electronic medical record. These features were comprised of standard sleep architecture measures (e.g. total sleep time and sleep onset latency), spectral features in specific frequency bands for each stage, and statistics of detected spindles, slow oscillations, and anatomical (across-lead) coherence. Overall performance of dementia classification was similar across the 3 model types with a max area under the ROC curve of 0.78, suggesting moderate fit.

The concept of model interpretability goes beyond performance—it includes an understanding of why the model arrived at the features that led to the output discrimination [4]. A strength of this article is that the authors investigated the relevance of the input sleep features on dementia discrimination using an odds ratio with false discovery rate-controlled analysis akin to genomic feature selection in large-scale omics studies. These model interpretability analyses revealed spectral-specific differences such as abnormal theta (4–8 Hz) and delta (0.5–4 Hz) activity in W/N1 stages or impaired spindles/slow oscillations in N2 sleep that were important for classifying dementia and MCI compared to cognitively normal. Features from the model interpretation by Ye et al. that were consistent with nonmachine learning studies of sleep include increased theta/alpha (8–12 Hz) power in W/N1 sleep in an AD case/control study [5]. Similarly, with regard to REM sleep, features of theta, sigma (11–15 Hz), and alpha spectral bands were top discriminative features for cognitively normal versus MCI and dementia, with REM sleep reduction and EEG slowing having been previously identified in patients with AD [6].

Another strength of this paper involves a clever approach to define the age-relationship of the top discriminative sleep features by taking a linear combination of disease class, age, and class times age interaction terms to explain each feature outcome. Here the intuition is that a sleep feature can change across age between cognitively normal and diseased classes in multiple ways. For example, N2 kurtosis, a statistical measure of distribution shape, can have similar slopes across disease classes but start out with different intercepts, while N1 theta/alpha ratio minimum can have entirely crossing slopes whose age-related relationship changes depending on the class membership at that age.

While the authors’ machine learning approach to predict dementia stage can be done in an automated and large-scale fashion which could in theory be applied to sleep study data acquired globally, it is important to recognize that sleep EEG features that predict dementia status may or may not be features that translate to future “dementia risk” in cognitively normal older individuals. That said, it is reassuring that some features that distinguish dementia in their model, such as reduced spindle density, are features that are also predictive of tau load by fluid biomarkers, reflecting dementia risk, in cognitively normal older individuals [7].

The authors’ findings should also be viewed with certain methodological limitations in mind. They employed analysis of clinical PSGs, which are performed for heterogeneous indications, including what is likely a majority of sleep apnea evaluations. Sleep apnea is highly prevalent in older adults [8] and may serve as a risk factor for dementia [9] through mechanisms such as hypoxemic burden [10] that may not have a clear EEG correlate. Additionally, the authors utilized both diagnostic and positive airways pressure (PAP) titration studies, potentially from the same individuals, in which marked changes to the EEG can exist as a function of sleep apnea treatment [11, 12]. The authors acknowledge that the “dementia” and “MCI” clinical labels were determined through chart review, and in fact, could reflect disparate underlying neuropathology, for example synucleinopathies versus tauopathies, that could have a distinct impact on sleep.

Another methodological limitation is that the authors did not use an independently acquired PSG dataset to test the validity of their dementia classification models. Presumably, the use of over 1000 features may introduce issues of collinearity, particularly when restricting the machine learning model to a linear kernel. Relatedly, additional interpretability analyses of models that might one day be used clinically are of utmost importance and should proceed prior to models being used in clinical trials/diagnoses. For example, machine learning models whose predictions are evaluated for feature reduction, rule extraction, or local explanation provide a reasonable tradeoff between “black box” decisions and clinical reasoning that includes types of patient interaction not easily stored in the electronic medical record [13]. Additionally, the use of probability values for cognitive status based on multiple tests of dementia risk assessment, rather than dichotomized as 0/1 as in the case of most machine learning models, would perhaps increase the utility of the proposed approach.

In summary, the study by Ye et al. takes steps of interpretability characterization that move our field one step closer to employing types of machine learning models in clinical sleep research, like identifying those who may respond to early interventions of amyloid or tau therapy. In order to progress to this lofty goal, it is likely that additional sleep data, such as respiratory, cardiovascular, and limb movements, all might contribute to both improving dementia discrimination while reinforcing existing evidence for sleep and its disorders in AD research.

## References

[1] Ju YE , et al. Sleep and Alzheimer disease pathology—a bidirectional relationship. Nat Rev Neurol. 2014;10:115–119. doi:10.1038/nrneurol.2013.269 24366271 PMC3979317

[2] Cedernaes J , et al. Candidate mechanisms underlying the association between sleep-wake disruptions and Alzheimer’s disease. Sleep Med Rev. 2017;31:102–111. doi:10.1016/j.smrv.2016.02.002 26996255 PMC4981560

[3] Ye EM , et al. Dementia detection from brain activity during sleep. Sleep. 2023;46(3). doi:10.1093/sleep/zsac286 PMC9995788 36448766

[4] Murdoch WJ , et al. Definitions, methods, and applications in interpretable machine learning. Proc Natl Acad Sci U S A. 2019;116:22071–22080. doi:10.1073/pnas.1900654116 31619572 PMC6825274

[5] Fahimi G , et al. Index of theta/alpha ratio of the quantitative electroencephalogram in Alzheimer’s disease: a case-control study. Acta Med Iran. 2017;502:506. 29034646

[6] Montplaisir J , et al. Sleep disturbances and eeg slowing in Alzheimer’s disease. Sleep Res Online. 1998;1:147–151. 11382871

[7] Kam K , et al. Sleep oscillation-specific associations with Alzheimer’s disease CSF biomarkers: novel roles for sleep spindles and tau. Mol Neurodegener. 2019;14:10. doi:10.1186/s13024-019-0309-5 30791922 PMC6385427

[8] Senaratna CV , et al. Prevalence of obstructive sleep apnea in the general population: a systematic review. Sleep Med Rev. 2017;34:70–81. doi:10.1016/j.smrv.2016.07.002 27568340

[9] Mullins AE , et al. Obstructive sleep apnea and its treatment in aging: effects on alzheimer’s disease biomarkers, cognition, brain structure and neurophysiology. Neurobiol Dis. 2020;145(10):5054. doi:10.1016/j.nbd.2020.105054 PMC7572873 32860945

[10] Kam K , et al. Acute OSA impacts diurnal Alzheimer’s biomarkers through nocturnal hypoxemia and state transitions. Am J Respir Crit Care Med.2022;206(8):1039–1042 doi:10.1164/rccm.202202-0262LE35696622

[11] D’Rozario AL , et al. Improvements in cognitive function and quantitative sleep EEG in OSA after six months of CPAP treatment. Sleep. 2022;45(6). doi:10.1093/sleep/zsac013 PMC9189957 35029691

[12] Parker JL , et al. The association between obstructive sleep apnea and sleep spindles in middle-aged and older men: a community-based cohort study. Sleep. 2022;45. doi:10.1093/sleep/zsab282 34850237

[13] Aria M , et al. A comparison among interpretative proposals for Random Forests. Mach Learn Appl. 2021;6:100094. doi:10.1016/j.mlwa.2021.100094

